# APP antisense oligonucleotides reduce amyloid-β aggregation and rescue endolysosomal dysfunction in Alzheimer’s disease

**DOI:** 10.1093/brain/awae092

**Published:** 2024-03-25

**Authors:** Christy Hung, Emre Fertan, Frederick J Livesey, David Klenerman, Rickie Patani

**Affiliations:** Human Stem Cells and Neurodegeneration Laboratory, The Francis Crick Institute, London NW1 1AT, UK; Developmental Biology and Cancer Department, UCL Great Ormond Street Institute of Child Health, Zayed Centre for Research into Rare Disease in Children, London WC1N 1DZ, UK; Department of Chemistry, University of Cambridge, Cambridge CB2 1EW, UK; Developmental Biology and Cancer Department, UCL Great Ormond Street Institute of Child Health, Zayed Centre for Research into Rare Disease in Children, London WC1N 1DZ, UK; Department of Chemistry, University of Cambridge, Cambridge CB2 1EW, UK; UK Dementia Research Institute at University of Cambridge, Cambridge CB2 0XY, UK; Human Stem Cells and Neurodegeneration Laboratory, The Francis Crick Institute, London NW1 1AT, UK; Department of Neuromuscular Diseases, Queen Square Institute of Neurology, University College London, London WC1N 3BG, UK

**Keywords:** endosome, lysosome, autophagy, aggregation, amyloid precursor protein, iPSCs

## Abstract

*APP* gene dosage is strongly associated with Alzheimer’s disease (AD) pathogenesis. Genomic duplication of the *APP* locus leads to autosomal dominant early-onset AD. Individuals with Down syndrome (trisomy of chromosome 21) harbour three copies of the *APP* gene and invariably develop progressive AD with highly characteristic neuropathological features.

Restoring expression of *APP* to the equivalent of that of two gene copies, or lower, is a rational therapeutic strategy, as it would restore physiological levels of neuronal APP protein without the potentially deleterious consequences of inadvertently inducing loss of APP function.

Here we find that antisense oligonucleotides (ASOs) targeting *APP* are an effective approach to reduce APP protein levels and rescue endolysosome and autophagy dysfunction in *APP* duplication and Trisomy 21 human induced pluripotent stem cell (hiPSC)-derived cortical neurons. Importantly, using ultrasensitive single-aggregate imaging techniques, we show that APP targeting ASOs significantly reduce both intracellular and extracellular amyloid-β-containing aggregates.

Our results highlight the potential of *APP* ASOs as a therapeutic approach for forms of AD caused by duplication of the *APP* gene, including monogenic AD and AD related to Down syndrome.

## Teaser

APP gene dosage is strongly associated with Alzheimer’s disease pathogenesis. Hung *et al.* show that antisense oligonucleotides targeting APP can reduce APP protein levels and rescue impaired protein clearance in human cortical neurons with APP duplication, and can reduce both intracellular and extracellular Aβ-containing aggregates.

## Introduction

Alzheimer’s disease (AD) is a progressive neurodegenerative disease and the most common cause of dementia. Accumulation of extracellular amyloid-β (Aβ) peptide fragments of the APP protein and intraneuronal neurofibrillary tangles of the microtubule-associated protein tau are the cellular hallmarks of AD. *APP* gene dosage is strongly associated with AD pathogenesis. Rare *APP* promoter mutations that significantly increase APP expression levels are associated with early-onset AD.^1^ Mild (∼20%) *APP* overexpression is sufficient to increase AD risk in the general population.^2^ Genomic duplication of the *APP* locus leads to autosomal dominant early-onset AD.^3^ Similarly, individuals with Down syndrome (trisomy of chromosome 21) harbour three copies of the *APP* gene and typically develop progressive AD with characteristic neuropathological features including amyloid plaques, neurofibrillary tangles and neuronal loss. Conversely, a missense mutation (A673T) in *APP* that results in a decrease in APP cleavage by β-secretase protects against AD and age-related cognitive decline.^4^ These findings strongly implicate overexpression of APP protein as one pathomechanism of AD.

Dysfunction of the endolysosomal-autophagy network is emerging as an important pathogenic process in AD. In mouse models of AD, faulty lysosomal acidification and autophagy dysfunction develop well before the appearance of extracellular amyloid plaque deposition.^5^ Misaccumulation of lysosomal dense bodies surrounding amyloid plaques has also been observed in post-mortem AD brain samples.^6^ Enlargement of Rab5+ early endosomes in Down syndrome occurs as early as in the fetal period.^7^ Recent genome-wide association studies (GWASs) have identified single nucleotide polymorphisms in several genes with roles in the endolysosomal and autophagy systems as impacting disease risk, which further underscores the significance of the endolysosomal and autophagy systems in disease pathogenesis.

We and others have shown that the accumulation of APP protein fragments generated from the amyloidogenic pathway within endosomal compartments disrupts endolysosomal function.^6-12^ We reported previously that increased *APP* gene dosage and *APP* mutations lead to early and late endosome enlargement, lysosome dysfunction and autophagy impairment in human induced pluripotent stem cell (hiPSC)-derived neurons.^8^ Our data suggest that reducing expression of *APP* to the equivalent of that of two gene copies, or lower, is a rational therapeutic strategy.

It has been previously established that antisense oligonucleotides (ASOs) targeting *APP* mRNA is an effective approach to reducing APP levels in *SORL1* KO human hiPSC-derived cortical neurons in a dosage-dependent manner, which corrects the endolysosomal defects present in these neurons.^9^ We sought to address if the same approach might restore physiological levels of APP in human neurons carrying an extra copy of the *APP* gene. Through dose optimization, we show that *APP* ASOs are effective in normalizing *APP* transcript levels from what would be expected from three copies back down to the equivalent of would be transcribed from two copies (i.e. a ∼33% reduction to restore expression of *APP* to the equivalent of that from two gene copies, or lower). We further show that *APP* ASOs rescue endolysosome and autophagy dysfunction in human *APP* duplication neurons by restoring lysosomal acidity to physiological levels. Importantly, using ultrasensitive single-molecule imaging techniques, we show a significant reduction in both intracellular and extracellular (secreted) Aβ-containing soluble aggregates. Our results highlight the potential of *APP* ASOs as a therapeutic approach for forms of AD caused by duplication of the *APP* gene, including monogenic AD and Down syndrome-related AD.

## Materials and methods

### Experimental model and subject details

Control hiPSC lines: Non-Demented-Control (NDC), SFC840 (StemBANCC), and AD3.1 (StemBANCC). All AD lines were previously reported and characterized: *APP* duplication^13^ and Ts21 hiPSCs.^13^ This research was carried out in accordance with the UK Code of Practice for the Use of Human Stem Cell Lines.

### Directed differentiation to human cortical neuron culture

Directed differentiation of hiPSCs to cortical neurons was carried out as previously described.^13^ Briefly, dissociated hiPSCs were plated on six-well plates coated with GelTrex (Life Technologies) and neural induction was initiated by a culture medium that supports neurogenesis; specficially: a 1:1 mixture of N2- and B27-containing media (N2B27) (supplemented with 1 μM dorsomorphin and 10 μM SB431542 to inhibit TGFβ signalling during neural induction). Media was replaced every 24 h. At Day 12, neuroepithelial cells were harvested with dispase and replated in laminin-coated plates with FGF2-containing media for 4 days. FGF2 was then withdrawn and dissociated using Accutase and neural progenitor cells were plated on GelTrex-coated plates. Plated neurons were maintained for up to 120 days with a medium change every 2–3 days.

### Protein analysis

Extracellular Aβ_1–42_, Aβ_1–40_ and Aβ_1–38_ were measured in conditioned media using multiplexed MesoScale Discovery assays on a Quickplex SQ120 instrument (MesoScale Discovery) according to the manufacturer’s instructions. Conditioned media samples from experiments collected at different time points were frozen at −80°C.

### Protein extraction and western blot analysis

For immunoblotting, whole cell lysate protein was extracted with RIPA buffer (Sigma) supplemented with protease inhibitors (Sigma) and Halt phosphatase inhibitors (ThermoFisher Scientific). Protein quantification was performed using Precision Red Advanced Protein Assay buffer (Cytoskeleton, Inc.). Equal amounts of protein samples were then loaded onto a gel and separated on a 4–12% sodium dodecyl-sulfate polyacrylamide gel electrophoresis (SDS-PAGE) and transferred to polyvinylidene fluoride (PVDF) membranes. Samples were then blocked with Li-Cor blocking buffer at room temperature for 1 h followed by primary antibody incubation overnight at 4°C. For detection, membranes were incubated with species specific near infra-red fluorescent antibodies (IRDye, Licor) at room temperature for 1 h and imaged using a Li-Cor Odyssey system. Antibodies used in this study are listed in Supplementary Table 1.

### RNA extraction and nCounter gene expression assay

Total RNA was extracted using RNeasy Mini Kit according to the manufacturer’s protocol (QIAGEN). RNA concentration was measured by spectrophotometric analysis using a NanoDrop™ 2000/2000c spectrophotometer, and 100 ng total RNA were loaded for each nCounter assay. A probe set designed by NanoString Technologies was used to analyze gene expression for 770 genes from each sample. Assays were performed using the NanoString protocols per the manufacturer’s instructions.

### Image acquisition and analysis

For the assessment of *in vitro* CTSD enzyme activity and CTSB enzyme activity assay, neurons were incubated with BODIPY FL-Pepstatin A (BP) (Thermo Fisher Scientific, Cat. No. P12271) or Magic Red Cathepsin-B Assay Kit (Cat. No. ICT938) according to the manufacturer’s instructions for 30 min. Subsequently, live-cell imaging was performed using a Zeiss SP8 confocal microscope within a CO_2_ environment chamber.

For immunostaining, cells were fixed in 4% paraformaldehyde (PFA) in phosphate buffered saline (PBS) followed by permeabilization with 0.3% Triton X-100 (Sigma). Fixed cells were blocked with 10% normal goat serum (Sigma) in PBS, probed with primary antibodies diluted in blocking solution and detected with goat anti-mouse, anti-chicken or anti-rabbit secondary antibodies coupled to Alexa Fluor 488, 594 or 647. Images were acquired using either a Zeiss SP8 confocal microscope or an instant structured illumination microscope (×150 oil objective with deconvolution).

For the analysis of vesicle size, Rab5+ and Lamp1+ puncta quantifications were done using Imaris software. Surface masks were created using the Tuj1 staining to indicate neurons.

### Antisense oligonucleotides

Antisense oligonucleotides (IDT) had the following modifications on a phosphorothioate (PS) backbone: a central gap region of ten 2′-deoxynucleotides flanked on both sides by five 2′-methoxyethyl nucleotides wings to improve nuclease resistance. All cytidine residues within the central gap region are 5-methyl deoxycytidine. The ASOs were delivered to hiPSC-derived neurons by addition to extracellular media for a 10-day period. *APP* ASO sequence—TGTCACTTTCTTCAGCCAGT.^14^

### Single-molecule pull-down imaging

Single-molecule pull-down (SiMPull) coverslip preparation was performed as previously described.^15^ Briefly, neutravidin (0.2 mg/ml) in PBST (PBS with 0.05% Tween 20) was added to glass coverslips (26 × 76 mm, thickness 0.15 mm, Thermo Scientific) covalently mounted with polyethylene glycol (PEG) and biotin for 10 min, followed by two wash steps with 0.05% PBST and once with 1% PBST. Afterwards biotinylated 6E10 (BioLegend, Cat. No. 803007, 10 nM) was added for 15 min, followed by two wash steps with 0.05% PBST and once with 1% PBST. The samples (either conditioned media or cell lysates) were added overnight at 4°C (for conditioned media) or at 20°C (for cell lysates) followed by two wash steps with 0.05% PBST and once with 1% PBST. The coverslips were then incubated with labelled AF647 labelled 6E10 (BioLegend, Cat. No. 803020, 500 pM) for 45 min, followed by three washing steps with 0.05% PBST.

To determine the number of fluorescent molecules in each image, a z-stack was generated in ImageJ. The images were cropped to 380 × 380 pixels and the contrast was adjusted. Using the negative control as a baseline, a threshold was applied to all images, and single molecules above this threshold were counted.

### Quantification and statistical analysis

Unless otherwise specified, statistical analysis was performed using GraphPad Prism (Version 9). Student’s *t*-test was used to compare differences between two groups. Significance threshold was defined as *P* < 0.05. Data are presented as mean ± standard error of the mean. Unless otherwise stated, data shown are calculated from biological replicates (*n* = 3 control lines and 3 *APP* duplication lines).

## Results

### *APP* ASOs restore physiological expression of neuronal APP protein levels in human cortical neurons

**Figure 1:**
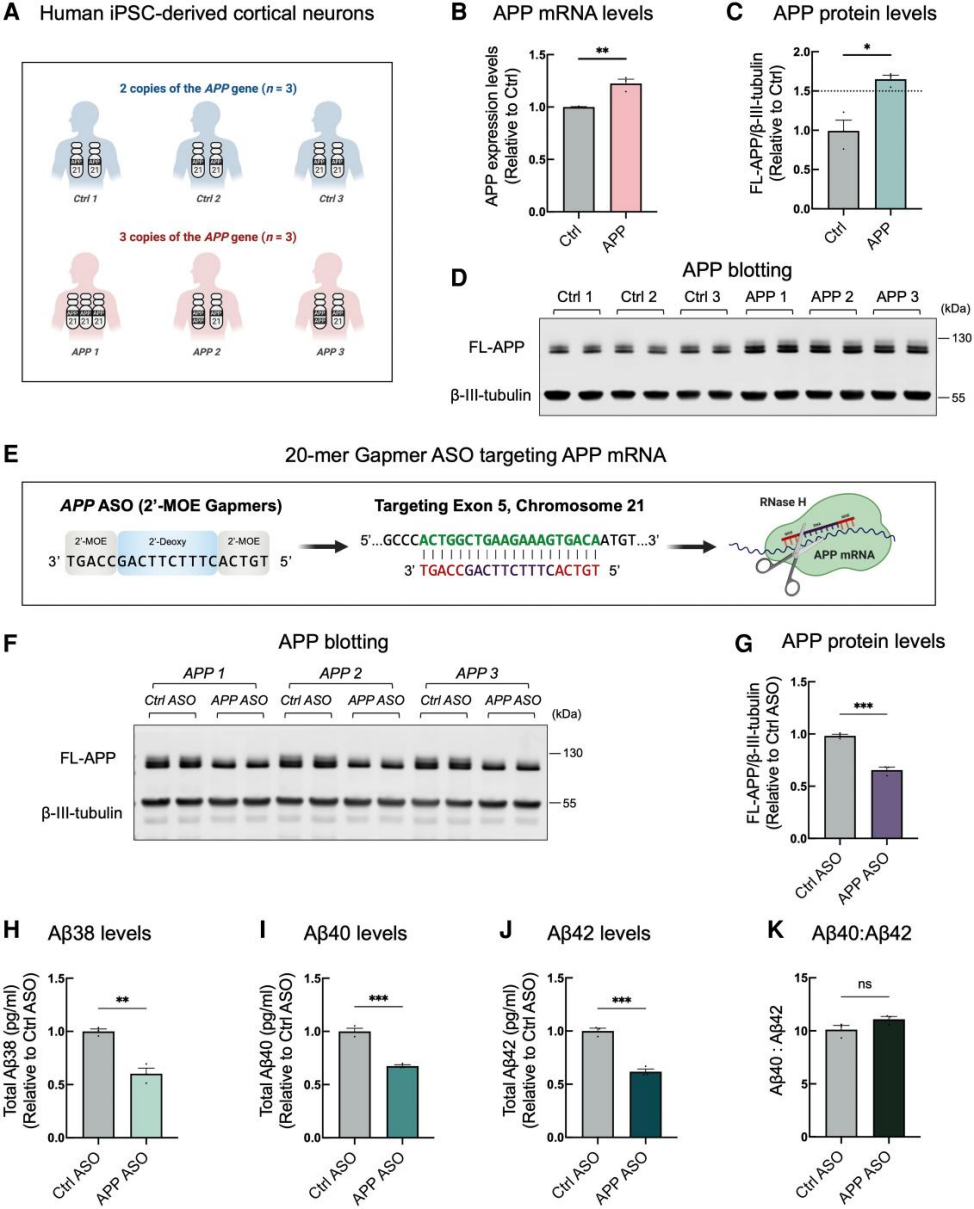
**Extracellular administration of *APP* antisense oligonucleotides restore physiological expression of neuronal APP protein levels in human induced pluripotent stem cell (hiPSC)-derived cortical neurons**. (**A**) Schematic of the cell lines used in this study. (**B**–**D**) *APP* mRNA (**B**) and full-length APP protein levels (**C**) were significantly higher in neurons generated from hiPSCs with three copies of *APP* than those detected in three independent controls. Representative western blots of full-length APP and neuron-specific β3-tubulin in control and APP neurons are shown (**D**). Levels of *APP* mRNA and full-length APP were calculated relative to those of controls (*n* = 3 control and 3 APP duplication lines). Data are represented as mean ± standard error of the mean (SEM). (**E**) Schematic of the binding region of *APP* antisense oligonucleotides (ASOs) and the experimental design; neurons were treated from Day 65 to Day 75 post-neural induction with *APP* ASOs. (**F** and **G**) Representative western blots of APP and neuron-specific β3-tubulin in *APP* duplication neurons treated with either control or *APP* ASO are shown in **F**. Levels of APP-FL (**G**) were calculated relative to β3-tubulin (*n* = 3 APP duplication lines). Data are represented as mean ± SEM. (**H**–**K**) *APP* neurons treated with *APP* ASO exhibit a significant decrease in the production of amyloid-β (Aβ)_38_ peptides (**H**), Aβ_40_ peptides (**I**) and Aβ_42_ peptides (**J**), with no change in the relative amounts of Aβ_40_ to Aβ_42_ (**K**) (*n* = 3 *APP* duplication lines). Data are represented as mean ± SEM. *P < 0.05, **P < 0.01, ***P < 0.001, ns = not significant.

We first generated cortical neurons from hiPSCs derived from three independent control individuals (‘control neurons’ hereafter) and three independent patient lines with an extra copy of *APP* gene (two *APP* duplication lines and one trisomy 21 hiPSC line; referred to as ‘*APP* neurons’ hereafter) (see Fig. 1A and Supplementary Table 2 for details of the cell lines). HiPSC-derived cortical neurons were generated using previously established methods.^13^ At Day 80 of differentiation, total RNA was harvested to analyze gene expression using the NanoString nCounter platform, as previously described.^16^ Gene expression levels were normalized against the geometric mean of 10 housekeeping genes. Both control and *APP* neurons expressed high levels of neuronal markers (*DLX1*, *DLX2*, *MAP2*, *MAPT*, *NEFL* and *ISLR2)* but relatively low expression levels of markers associated with other CNS cell types, such as astrocytes, microglia and oligodendrocytes (Supplementary Fig. 1A), confirming their robust differentiation into neurons. Cultures were also fixed and immunolabelled for neuronal markers (TUJ1 and MAP2), which showed similar cell density (Supplementary Fig. 1B). Consistent with previous studies,^8,13^ we found that increased *APP* gene dosage increases *APP* mRNA (Fig. 1B), full-length APP protein levels (Fig. 1C and D) and the production of extracellular Aβ_38_, Aβ_40_ and Aβ_42_ peptides in hiPSC-derived cortical neurons (Supplementary Fig. 1C–G).

We next questioned whether we can restore the physiological expression of neuronal APP protein levels in *APP* neurons using ASOs. To do so, we treated *APP* neurons with either a 20-mer gapmer control ASO or *APP* ASO targeting Exon 5 of the *APP* mRNA. These ASOs comprise a central gap region of ten 2′-deoxynucleotides, flanked on both sides by five 2′-methoxyethyl nucleotide wings with a phosphorothioate backbone (Fig. 1E). This specific ASO has been shown to effectively target the *APP* mRNA.^9,14^ In a published patent, a total of 37 gapmer ASOs, targeting various regions of the *APP* mRNA, were screened,^14^ and we employed the one that demonstrated the highest knockdown efficiency. Importantly, this ASO has previously been shown to effectively knockdown APP protein levels in a dosage dependent manner in human *SORL1* KO cortical neurons.^9^

ASOs were administered to neurons by gymnosis for a 10-day period. To visualize the uptake of APP ASOs, we employed fluorescein-labelled (6-FAM) *APP* ASOs and performed live-cell imaging. We found that nearly all hiPSC-derived cortical neurons contain *APP* ASOs after 24 h (Supplementary Fig. 2). Through dose optimization (Supplementary Fig. 3), we chose to use ASOs at the concentration of 1.75 μM to reduce APP protein levels by 33%. The control ASO possesses the same nucleotide composition as the *APP* ASO but in a different sequence, thus enabling us to distinguish between sequence-specific silencing and any potential non-specific effects in the disease neurons. Quantitative PCR analysis showed no significant changes in the expression levels of genes predicted to have two mismatches with the sequence of the *APP* ASO (Supplementary Fig. 4A). Using the NanoString nCounter platform to analyse the differential expression of over 770 genes after treatment with either the control or *APP* ASO, we observed that *APP* is the most significantly downregulated gene (Supplementary Fig. 4B).

We observed a significant reduction in APP levels in neurons treated with *APP* ASOs (Fig. 1F and G). This finding was supported by a significant decrease in the production of extracellular Aβ peptides as determined by multiplexed Aβ ELISAs (Fig. 1H–J), which included Aβ_38_, Aβ_40_ and Aβ_42_, with no change in the relative ratio of Aβ_40_ to Aβ_42_ (Fig. 1K).

### *APP* ASOs rescue early and late endosome/lysosome enlargement caused by increased *APP* gene dosage

Increased *APP* gene dosage leads to endolysosomal and autophagy dysfunction in hiPSC-derived neurons, as demonstrated through live-cell imaging of GFP or RFP fusion protein overexpression.^8^ To bypass any potential limitations of such overexpression, including possible changes in the morphology of organelles, we used super-resolution instant structured illumination microscopy (iSIM) to measure the number and size of endogenous Ras-related protein, Rab5 (a well-established early endosome protein) and lysosomal-associated membrane protein 1, LAMP1 (one of the most abundant lysosomal components). Consistent with previous studies,^8^ we observed a significant increase in the average size of early endosomes (Rab5+ puncta) and late endosomes/lysosomes (LAMP1+ puncta) in neurons generated from hiPSCs carrying an extra copy of the *APP* gene compared with control counterparts (Supplementary Fig. 5A–F). Distribution analysis of sizes revealed a significant increase in the frequency of larger puncta (>0.9 μm^2^) in *APP* neurons compared with controls (Supplementary Fig. 5A–F). We also observed a significant reduction in autophagy flux (Supplementary Fig. 5G and H). These data are together consistent with previous findings that increased *APP* gene dosage results in early endosome and late endosome/lysosome enlargement, lysosomal dysfunction and autophagy impairment in hiPSC-derived neurons.^8^

**Figure 2:**
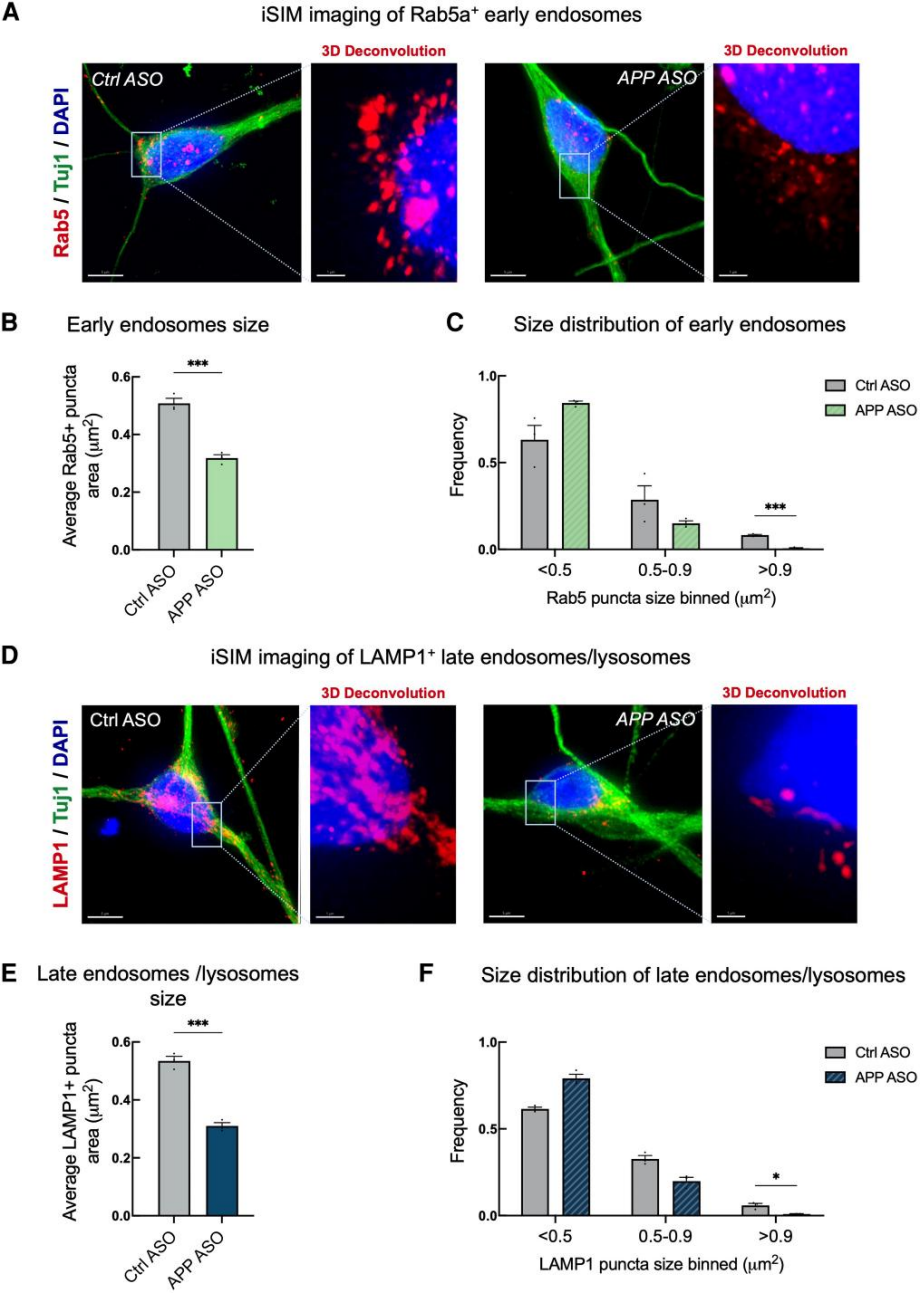
***APP* antisense oligonucleotides rescue early endosome and late endosome/lysosome enlargement caused by increased *APP* gene dosage.** (**A**) Representative immunocytochemistry of human induced pluripotent stem cell (hiPSC)-derived neurons expressing Rab5 proteins. Scale bars = 5 μm. (**B** and **C**) A significant decrease in the average size of early endosomes (**B**) and frequency (**C**) of early endosomes with size > 0.9 μm^2^ in *APP* neurons treated with *APP* antisense oligonucleotides (ASOs) compared with control ASOs (*n* = 3 APP duplication lines, each in technical triplicate). Data are represented as mean ± standard error of the mean (SEM). (**D**) Representative immunocytochemistry of hiPSC-derived neurons expressing LAMP1 proteins. Scale bars = 5 μm. (**E** and **F**) A significant decrease in the average size of late endosomes/lysosomes (**E**) and frequency (**F**) of LAMP1+ puncta with size > 0.9 μm^2^ in *APP* neurons treated with *APP* ASOs compared to control ASOs (*n* = 3 *APP* duplication lines, each in technical triplicate). Data are represented as mean ± SEM. *P < 0.05, ***P < 0.001.

Against this background, we hypothesized that restoring the physiological expression of neuronal APP protein will rescue endolysosomal dysfunction in *APP* neurons. We used super-resolution iSIM to measure the size of endogenous Rab5 in *APP* neurons treated with *APP* ASOs. A 10-day treatment with *APP* ASOs restored the average size of Rab5-positive endosomes to the levels seen in control neurons (Fig. 2A–C compared with Supplementary Fig. 5A). To investigate this further, we also measured the size of endogenous LAMP1 (Fig. 2D–F). We found a significant reduction in the size of LAMP1-positive late endosomes/lysosomes in *APP* ASOs treated neurons. Importantly, control ASOs did not alter the sizes of either Rab5+ early endosomes or LAMP1+ late endosomes/lysosomes, showing the specificity of the *APP* ASOs for the endolysosomal pathology (Supplementary Fig. 6).

### *APP* ASOs restore lysosomal acidity and rescue autophagy dysfunction in *APP* neurons

**Figure 3:**
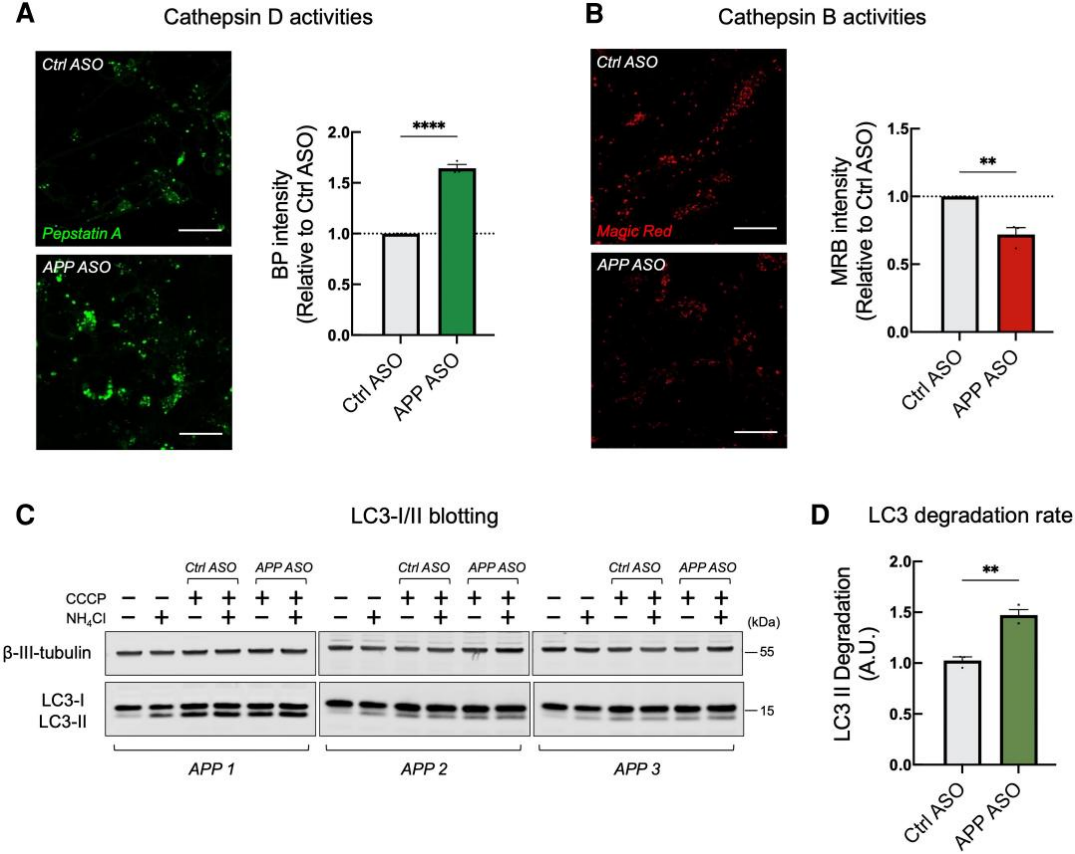
***APP* antisense oligonucleotides restore lysosomal acidity and rescue autophagy dysfunction in APP neurons.** (**A**) Representative images (*left*) and quantification (*right*) of BODIPY FL-Pepstatin A (BP) labelling in *APP* duplication neurons treated with control or *APP* antisense oligonucleotides (ASOs) (*n* = 3 APP duplication lines, each in technical triplicate). Scale bars = 10 μm. Data are represented as mean ± standard error of the mean (SEM). (**B**) Representative images (*left*) and quantification (*right*) of Magic Red cathepsin B substrate (MRB) labelling in *APP* duplication neurons treated with control or *APP* ASOs (*n* = 3 APP duplication lines, each in technical triplicate). Scale bars = 10 μm. Data are represented as mean ± SEM. (**C** and **D**) Autophagosome degradation was significantly increased in *APP* duplication neurons treated with *APP* ASOs, as calculated from the western blot analysis. Representative western blots of LC3 I/II and neuron-specific β3-tubulin are shown (**C**). Autophagosome degradation following autophagy induction with CCCP (20 μM) in the absence or presence of NH_4_Cl was calculated (**D**) (*n* = 3 APP duplication lines, each in technical triplicate). Data are represented as mean ± SEM. **P < 0.01, ****P < 0.0001.

To further examine whether the reduction in APP protein also rescued lysosomal and autophagy dysfunction, we first measured the level of activation of cathepsin D, a major lysosomal aspartyl protease optimally active at low pH, using BODIPY FL-Pepstatin A (BP). BP is an affinity reagent that binds to the enzymatically active form of cathepsin D. We observed a significant increase in the intensity of BP fluorescence in neurons treated with *APP* ASOs, indicating a restoration in the level of lysosomal cathepsin D activity (Fig. 3A).

Given the role of an extra *APP* gene copy in causing lysosomal acidification deficits in DS,^17^ we hypothesized that *APP* ASOs may rescue cathepsin D activity by restoring normal lysosomal acidity. Cathepsin B is a lysosomal protease that is stable and optimally active at a higher pH range (approximately 6.0–6.5).^18^ Consistent with our hypothesis, neurons treated with *APP* ASOs exhibited a significantly lower cathepsin B activity using Magic Red cathepsin B substrate (Fig. 3B), a membrane permeable cathepsin B target sequence peptide that generates red fluorescence upon enzymatic cleavage.

Given the dynamic nature of the lysosomal and autophagy pathway, we also measured autophagic flux using the same methods as used in Supplementary Fig. 5G. Autophagy was induced in both control and *APP* neurons by CCCP treatment, as demonstrated by an upregulation of LC3-II in the presence of both CCCP and NH_4_Cl compared with neurons treated with NH_4_Cl alone (Fig. 3C and Supplementary Fig. 5). However, we observed a further elevation of LC3-II levels only in neurons treated with *APP* ASOs, indicating a significant increase in the rate of autophagosome degradation (Fig. 3D and Supplementary Fig. 7). Together, our data suggest that *APP* ASOs rescued cathepsin D activity by restoring normal lysosomal acidity and ameliorated autophagy dysfunction in *APP* neurons.

### *APP* ASOs reduce intracellular and extracellular Aβ-containing aggregates

**Figure 4:**
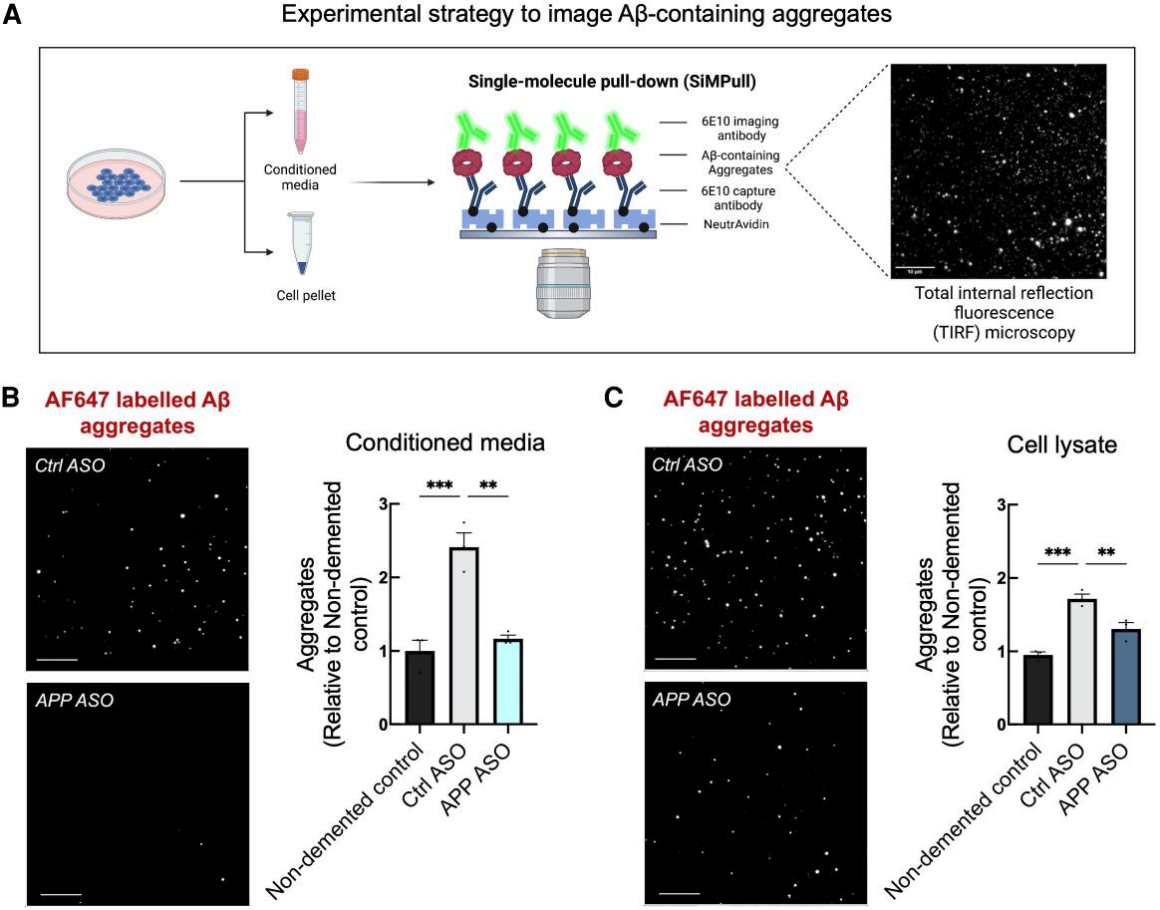
***APP* antisense oligonucleotides reduce intracellular and the secretion of extracellular amyloid-β-containing aggregates.** (**A**) Schematic of single-molecule pull-down (SiMPull) technique. In this assay, amyloid-β (Aβ)-containing aggregates are captured using a surface-tethered 6E10 antibody and imaged using total internal reflection fluorescence (TIRF), after adding primary detector antibodies for Aβ (Alexa-Fluor-647-labelled 6E10). Scale bars = 10 μm. (**B** and **C**) Analysis of conditioned media (**B**) and cell lysate (**C**) using SiMPull revealed a statistically significant difference in the number of Aβ-containing aggregates in *APP* duplication neurons treated with *APP* antisense oligonucleotides (ASOs) (*n* = 3 *APP* duplication lines, each in at least two technical replicates). Scale bars = 10 μm. Data are represented as mean ± standard error of the mean. **P < 0.01, ***P < 0.001.

Accumulation of extracellular Aβ aggregates comprising Aβ peptide oligomers is considered an important pathogenic process in AD. However, characterization of the aggregates secreted by hiPSC-derived neurons is challenging due to their low concentrations and sub-diffraction limit size. We, therefore, used SiMPull to measure intracellular and extracellular (secreted) Aβ-containing aggregates. In this assay (Fig. 4A), Aβ-containing aggregates are captured using a surface-tethered 6E10 antibody and imaged using total internal reflection fluorescence (TIRF), after adding primary detector antibodies for Aβ (Alexa-Fluor-647-labelled 6E10).^15^ Using the same monoclonal antibody to sandwich Aβ aggregates renders unreacted monomers undetectable because they only contain one epitope. Analysis of conditioned media using SiMPull revealed a statistically significant decrease in aggregate number in *APP* neurons treated with *APP* ASOs to a level comparable to that of control neurons (Fig. 4B). We also observed a statistically significant reduction in the number of intracellular Aβ-containing aggregates (Fig. 4C). Together, our results show that APP ASOs are effective in reducing both intracellular and extracellular Aβ-containing aggregates in hiPSC-derived neurons.

## Discussion

AD is a major global health problem. The presence of plaques containing Aβ peptide fragments of the APP protein is one of the cellular hallmarks of AD. The amyloid hypothesis proposes that *APP* gene dosage is strongly associated with AD pathogenesis. An additional copy of the *APP* gene (duplication) is sufficient to cause autosomal dominant early-onset AD.^3^ Similarly, individuals with Down syndrome (trisomy of chromosome 21) harbour three copies of the *APP* gene and invariably develop progressive AD with characteristic neuropathological features including amyloid plaques, neurofibrillary tangles and neuronal loss.^19^ In this study, we first confirm previous findings that increased *APP* gene dosage increases *APP* mRNA, protein levels and Aβ production in hiPSC-derived cortical neurons.^8,13^ Using super-resolution iSIM imaging, we further confirm that increased *APP* gene dosage causes endogenous early and late endosome enlargement, lysosome dysfunction and autophagy impairment in *APP* neurons. The main finding of our work is that restoring the physiological expression of neuronal APP protein through ASOs rescues these APP-dependent phenotypes. Importantly, we show that *APP* ASOs can reduce both intracellular and extracellular Aβ-containing aggregates in human neurons, indicating that this approach bears potential therapeutic significance in forms of AD caused by duplication of the *APP* gene, including monogenic AD and AD related to Down syndrome.

AD is a multi-factorial complex disease that likely requires a polytherapeutic approach starting at the earliest disease stage.^20^ The majority of therapies for AD have been focused on reducing different forms and conformational states of extracellular Aβ proteins by monoclonal antibody-mediated immunotherapies.^21^ However, noting that different Aβ species have been observed in the brains of AD patients, it is not clear which of these species are neurotoxic, thereby potentially limiting the therapeutic benefit of these approaches.^22^ Compared to traditional immunotherapies, ASOs directed at mRNA appear to be an appealing alternative. Firstly, they can be delivered to the brain relatively efficiently, for example, intrathecal delivery of ASO that modulates the splicing of *SMN2* RNA for ventral spinal motor neurons have been successfully developed to treat spinal muscular atrophy (SMA).^23^ Furthermore, ASO-based therapies enable multi-targeting and can be adjusted sequentially to the disease stage.^20^ ASOs targeting mRNA or miRNA can be used not only to reduce expression, but also enhance it, thereby permitting the removal of malfunctioning proteins or the restoration of those with proper function. Additionally, ASOs offer the unique ability to target specific protein isoforms resulting from alternative splicing.^20^ For example, we previously reported that 3R to 4R *MAPT* splice-switching ASOs are sufficient to recapitulate molecular phenotypes of *VCP*-related FTD in control cortical neurons.^24,25^

*APP* ASOs can potentially overcome the limitations of traditional immunotherapies by targeting *APP* mRNA, thereby reducing full-length APP proteins and all forms of Aβ species. Prior elegant work that motivated our study includes the development of several ASOs aimed at lowering levels of Aβ either by targeting *APP* mRNA or its enzymes involved in amyloidogenic processing. For example, OL-1, an ASO targeting the *APP* mRNA region corresponding to the 17–30 amino acid fragment of Aβ, lowered APP expression in AD mouse models Tg2576 (APPswe) and SAMP8 mice.^26,27^ Another important study utilised a splice-switching ASO that causes skipping of the *APP* exon that encodes proteolytic cleavage sites required for Aβ peptide production.^28^ This study successfully demonstrated that ASO treatment in this context can lead to a reduction in Aβ_42_ levels in the hippocampus of wild-type C57BL/6J mice. Similarly, Alnylam Pharmaceuticals has submitted a clinical trial application for ALN-APP, an investigational RNAi therapeutic in development for the treatment of AD and cerebral amyloid angiopathy.

Our findings that *APP* ASOs can restore physiological expression of neuronal APP protein in human cortical neurons have potential important therapeutic implications across neurodegenerative disorders more broadly. The dysregulation of gene dosage due to duplication or haploinsufficiency involving dosage-sensitive genes is a major cause of multiple neurodegenerative diseases, such as the peripheral myelin protein 22 (*PMP22*) gene for Charcot-Marie-Tooth disease type 1A and the alpha-synuclein (*SNCA*) gene for Parkinson’s disease. Most therapies have targeted downstream pathways to ameliorate consequences of protein dysfunction. However, a therapy aimed at correcting the abnormal gene expression and therefore the overproduction of the toxic protein is an effective approach that will directly target the source of dysfunction. Our data show that ASOs are ideal candidates for such therapy, through their established mechanism of action; namely heterodimerization with target mRNAs by Watson-Crick base pairing and direct their catalytic degradation through the action of RNase H, or through RNase H independent mechanisms, resulting in reduced or modified protein expression.

## Supplementary Material

awae092_Supplementary_Data

## Data Availability

This paper reports no original code. Original data generated in this study are available from the corresponding authors upon request.
