# Supplementary material for: APP antisense oligonucleotides reduce amyloid-β aggregation and rescue endolysosomal dysfunction in Alzheimer’s disease
Source: Brain. 2024 Mar 25;147(7):2325–33. doi: 10.1093/brain/awae092 (PMC11224613; doi:10.1093/brain/awae092)
Supplement: awae092_Supplementary_Data [file awae092_supplementary_data.pdf]

Supplementary information

Mouse anti-beta-Amyloid, 1-16	BioLegend
Mouse monoclonal anti-APP C-Terminal Fragment	BioLegend
Rabbit polyclonal anti-Tubulin β-3	BioLegend
Mouse monoclonal anti-Tubulin β-3	BioLegend
Mouse monoclonal anti-β-actin	Sigma
Rabbit polyclonal anti-LAMP1	abcam
Rabbit polyclonal anti-LC3B	Sigma
Chicken polyclonal anti-MAP2	abcam
Rabbit monoclonal anti-Rab5	Cell Signaling Technology

Supplementary Table 1. Antibodies used in this study.

Control/patient	Cell line name	Source	Age at sampling	Sex of cell	Cell type
Control 1	UCSD224i-NDC1-2	University of California - San Diego	86Y	M	Fibroblast of skin
Control 2	STBCi322-A	StemBANCC; Oxford; United Kingdom	36Y	F	Fibroblast of skin
Control 3	STBCi026-A	StemBANCC; Oxford; United Kingdom	67Y	F	Fibroblast of skin
APP 1	DS1-iPS4	Children's Hospital Boston; Boston; USA	1Y	M	Fibroblast of skin
APP 2	UCSD236i-APP1-1	University of California - San Diego	51Y	M	Fibroblast of skin
APP 3	UCSD240i-APP2-2	University of California - San Diego	60Y	F	Fibroblast of skin

Supplementary table 2. Details of the cell lines used in this study.

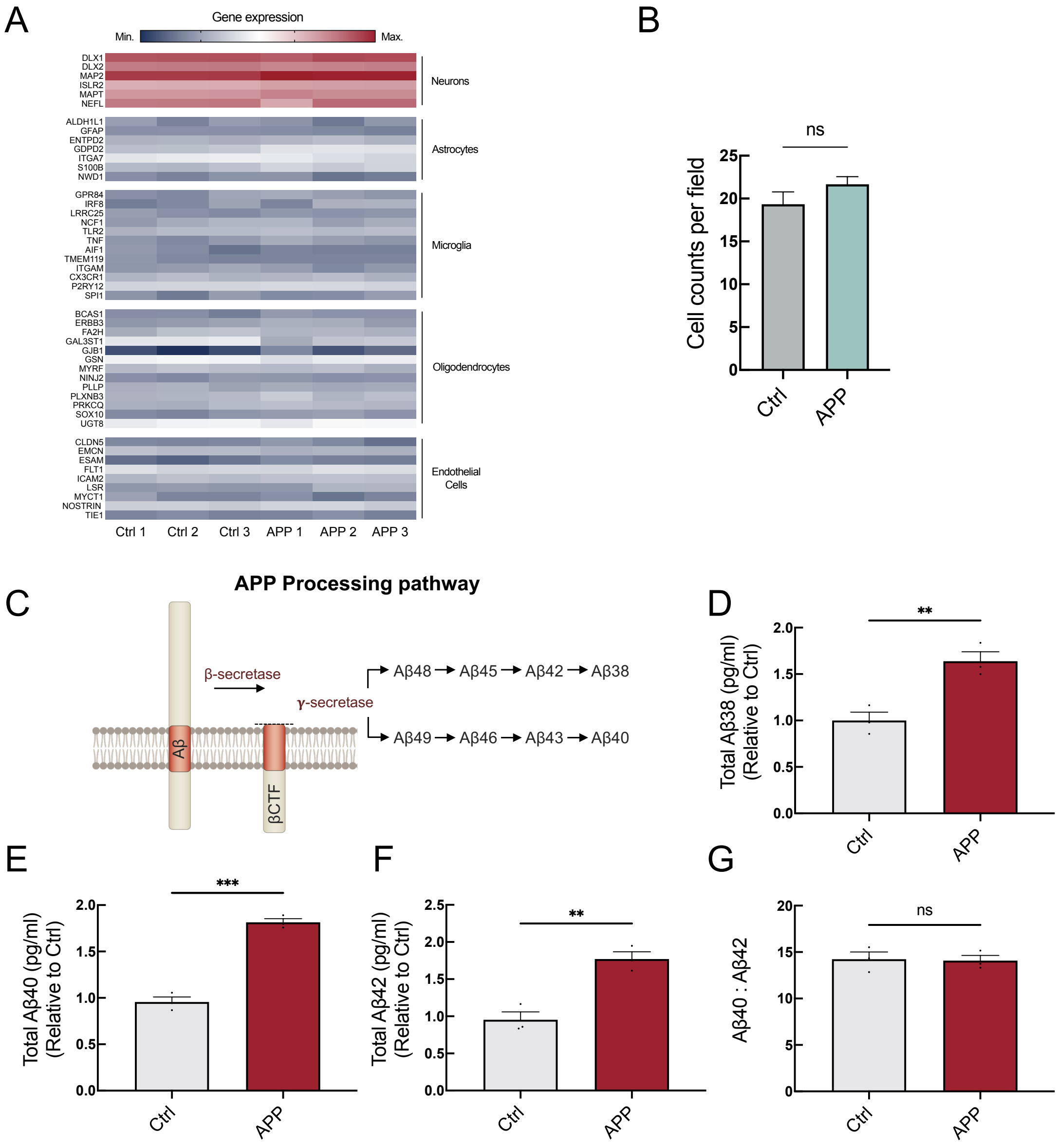

Figure S1. Increased *APP* gene dosage increases Aβ production in hiPSC-derived cortical neurons.

(A) Total RNA was harvested from day 80 cultures and analyzed using the NanoString nCounter platform. The heatmap shows the normalized expression levels of genes associated with cell-type specific markers, for both Control and APP cultures. Raw NanoString data were normalized to 10 housekeeping genes.
(B) Cell counts per field. Data represent mean ± SEM.
(C) Schematic of APP processing pathways.
(D – G) At 6 weeks in culture, APP neurons exhibit a significant increase in production of Aβ38 peptides (D), Aβ40 peptides (E) and Aβ42 peptides (F), with no change in the relative amounts of Aβ40 to 42 (G) (n = 3 control and 3 APP duplication lines, each in technical triplicate). Data represent mean ± SEM.

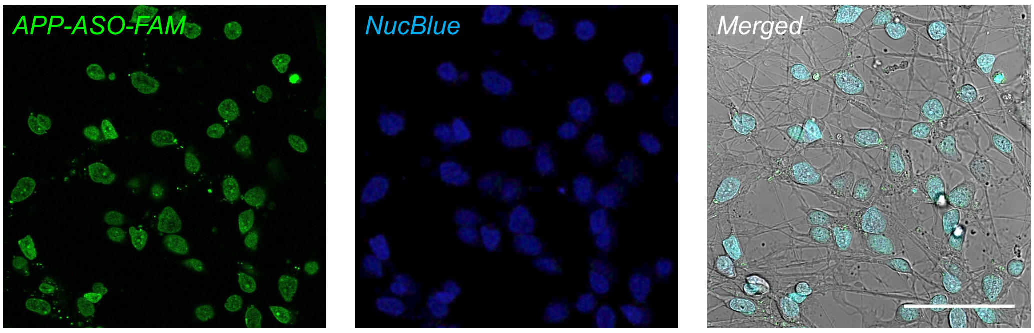

Figure S2. Efficient uptake of APP ASOs by hiPSC-derived cortical neurons after 24 hours.

Additional data to support the efficient uptake of APP ASOs by hiPSC-derived cortical neurons.

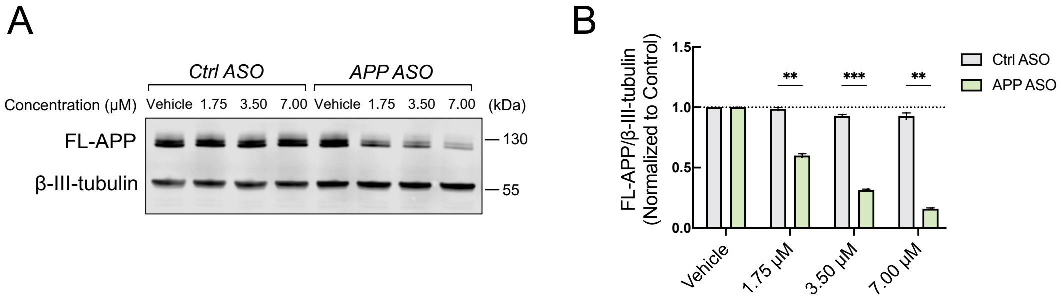

Figure S3. APP neurons treated with Ctrl or APP ASO exhibit a dose-dependent decrease in full-length APP protein. Representative western blots of APP and neuron-specific β3-tubulin in APP neurons treated with Ctrl or APP ASO are shown in (A). Levels of APP-FL (B) were calculated relative to β3-tubulin (n = 3).

A

Gene	Mismatch count	Mismatch annotation
LOC105375567	2	1: A>C, 7: C>A
FARP1	2	3: C>G,11: T>A
PHF14	2	15: T>G,17: G>T
MIR100HG	2	11: C>A,16: A>G

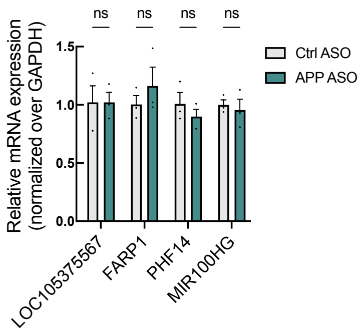

B

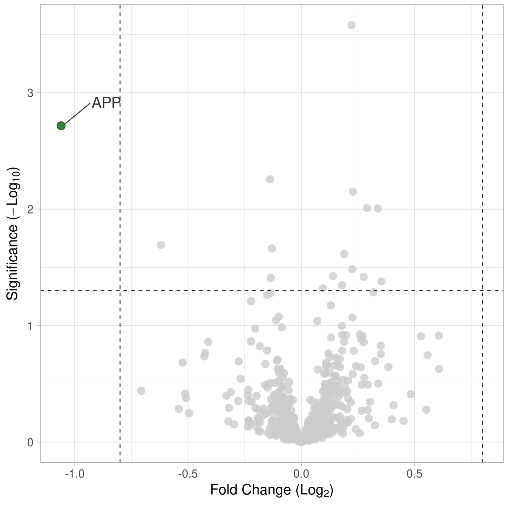

Figure S4. (A) No significant changes in the 4 RNA targets that have 2 mismatches with the APP ASO (n = 3 APP duplication lines treated with either control ASO or APP ASO). Data represent mean ± SEM. (B) Volcano plot showing that APP is the most significantly downregulated gene.

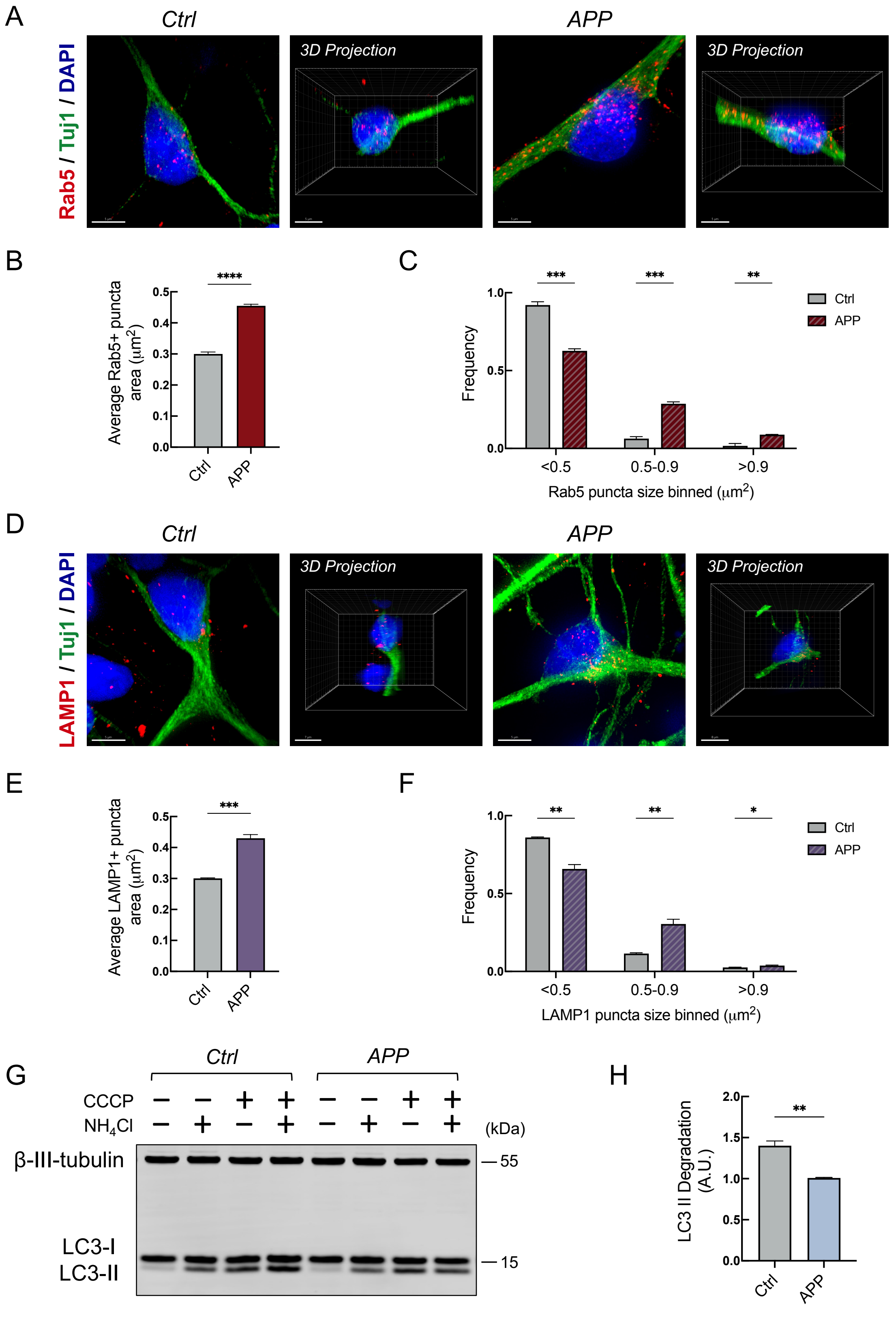

Figure S5. Increased *APP* gene dosage results in early and late endosome enlargement, lysosome dysfunction and autophagy impairment in hiPSC-derived neurons.

(A) Representative immunocytochemistry of hiPSC-derived neurons expressing Rab5 proteins (red, Rab5; green, β3-tubulin; blue, DAPI). Scale bars, 5 μm.
(B and C) A significant increase in the average size (B) and frequency of Rab5+ puncta with size > 0.9 μm^2(C) in *APP* neurons compared with controls (n = 3 control and 3 APP duplication lines, each in technical triplicate). Data represent mean ± SEM.
(D) Representative immunocytochemistry of hiPSC-derived neurons expressing LAMP1 proteins (red, LAMP1; green, β3-tubulin; blue, DAPI). Scale bars, 5 μm.
(B and C) A significant increase in the average size (B) and frequency of LAMP1+ puncta with size > 0.9 μm^2(C) in *APP* neurons compared with controls (n = 3 control and 3 APP duplication lines, each in technical triplicate). Data represent mean ± SEM.
(G and H) Autophagosome degradation was significantly reduced in *APP* duplication neurons compared with controls, as calculated from the western blot analysis. Representative western blots of LC3I/II and neuron-specific β3-tubulin from neurons derived from APP and Control hiPSCs are shown (G). Autophagosome degradation following autophagy induction with CCCP (20 μM) in the absence or presence of NH_4Cl was calculated (H) (n = 3 control and 3 APP duplication lines, each in technical triplicate). Data represent mean ± SEM.

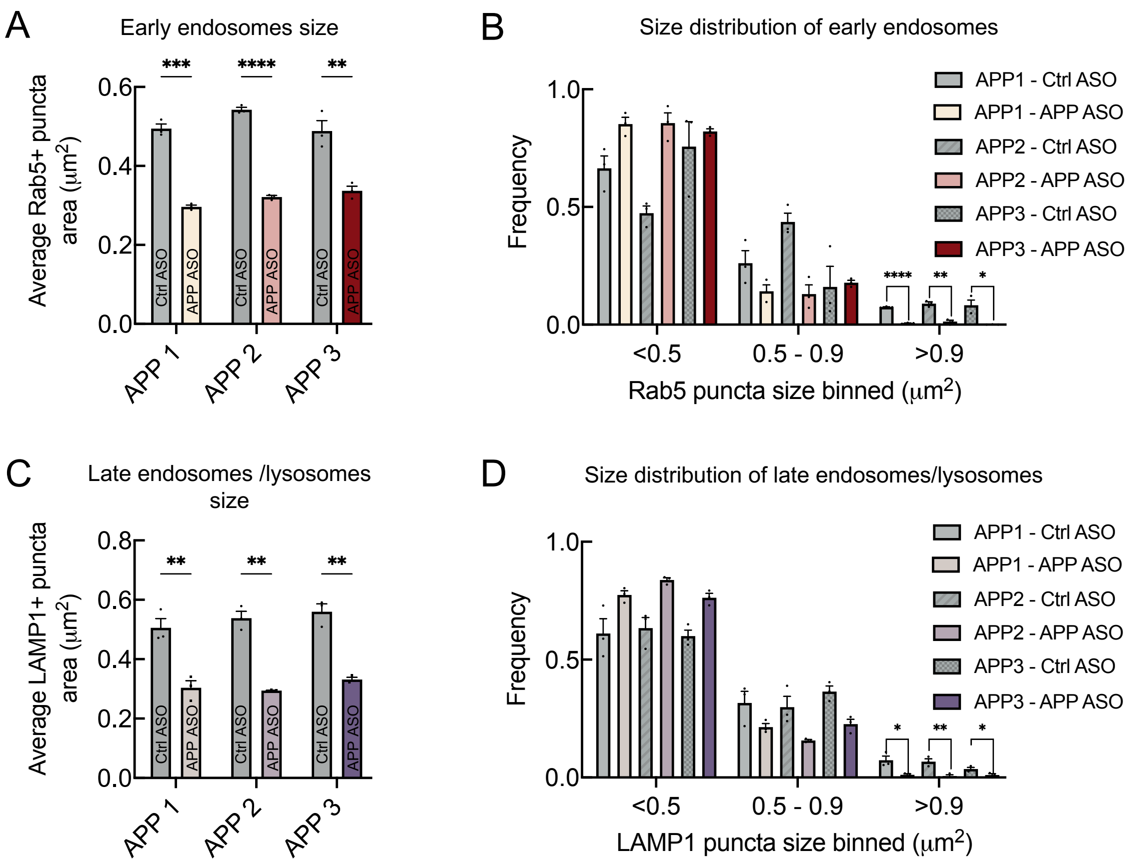

Figure S6. (A and B) A significant decrease in the average size of early endosomes (A) and frequency (B) of early endosomes with size > 0.9 μm^2 in APP neurons treated with APP ASOs compared to control ASOs. (C and D) A significant decrease in the average size of late endosomes/lysosomes (C) and frequency (D) of late endosomes/lysosomes with size > 0.9 μm^2 in APP neurons treated with APP ASOs compared to control ASOs (n = 3 independent biological inductions). Data represent mean ± SEM.

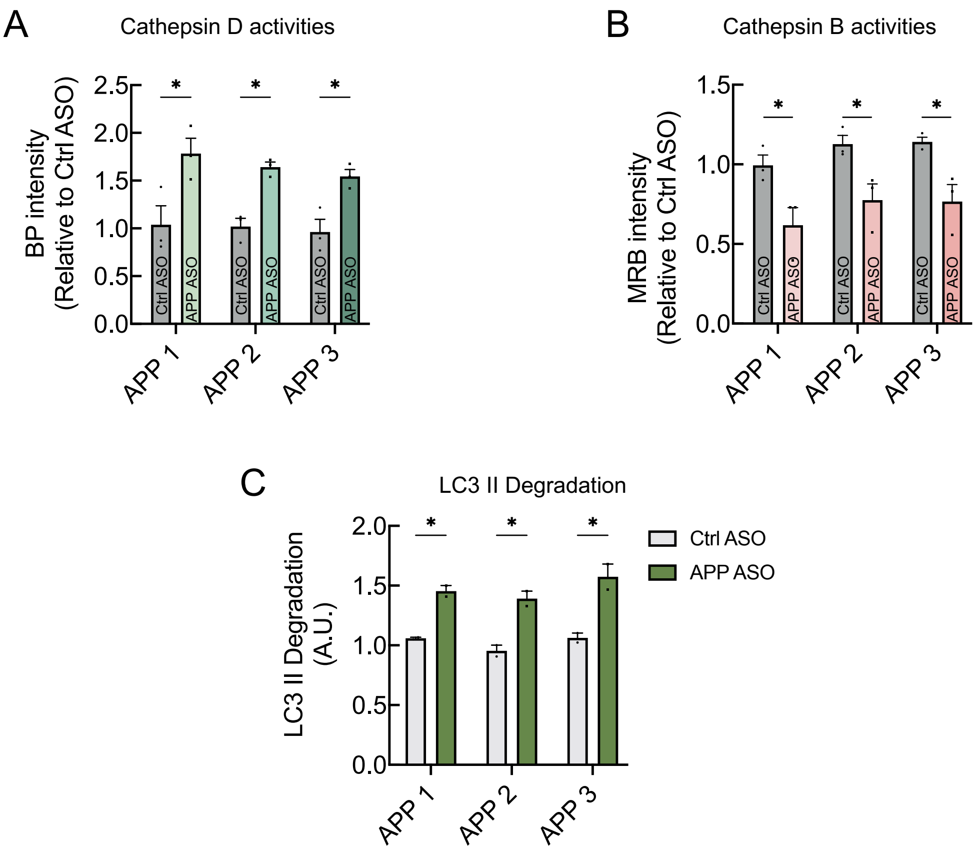

Figure S7. (A and B) Quantification of (A) BODIPY FL-Pepstatin A (BP) labelling and (B) Magic Red cathepsin B substrate (MRB) labelling in APP duplication neurons treated with control or APP ASOs. (C) Autophagosome degradation following autophagy induction with CCCP (20 µM) in the absence or presence of NH_4Cl was calculated. (n = 3 independent biological inductions). Data represent mean ± SEM.

Figure 1D

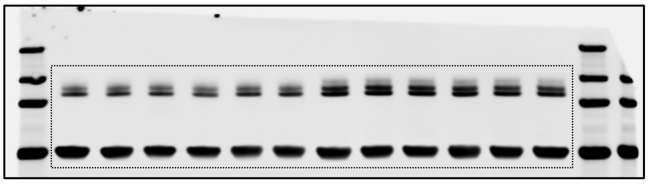

Figure 1F

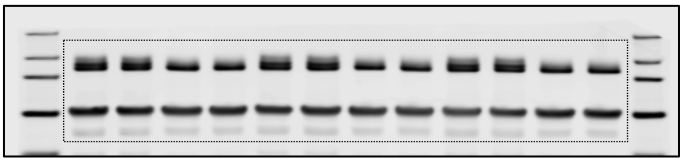

Figure 2C

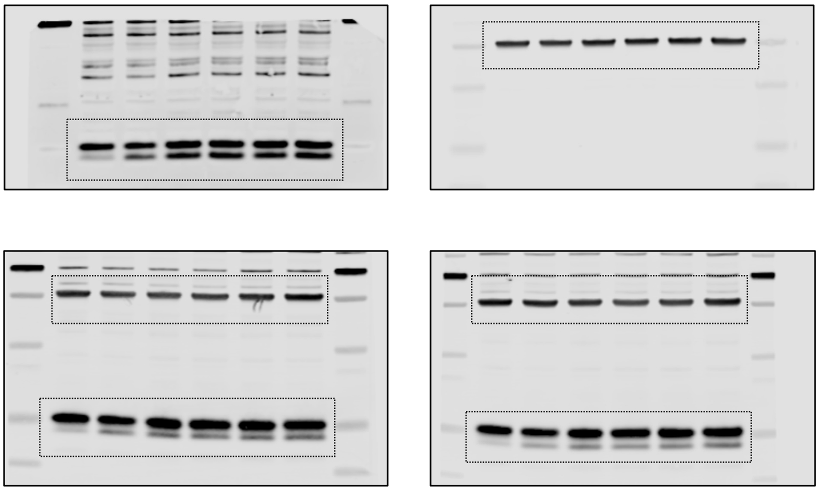

Figure S3A

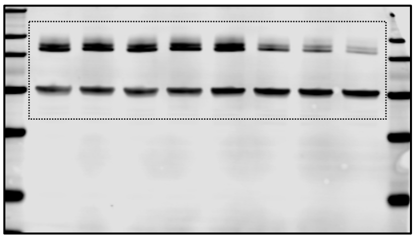

Figure S5G

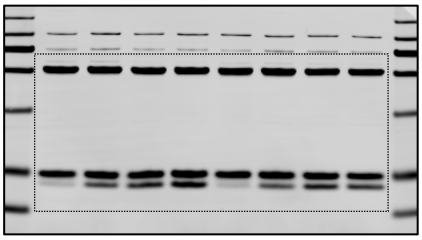

Figure S8. Uncropped western blots for Figure 1, 2 and Supplementary Figure 3 and 5. Dashed lines indicate bands used as representative images.
